# Copy number changes at 8p11-12 predict adverse clinical outcome and chemo- and radiotherapy response in breast cancer

**DOI:** 10.18632/oncotarget.24904

**Published:** 2018-03-30

**Authors:** Cathy B. Moelans, Caroline M. G. van Maldegem, Elsken van der Wall, Paul J. van Diest

**Affiliations:** ^1^ Department of Pathology, University Medical Center Utrecht, Utrecht, The Netherlands; ^2^ Department of Gynaecology, Albert Schweitzer Hospital, Dordrecht, The Netherlands; ^3^ Cancer Center, University Medical Center Utrecht, Utrecht, The Netherlands

**Keywords:** breast cancer, loss, prognostic, predictive, 8p

## Abstract

**Purpose:**

The short arm of chromosome 8 (8p) is a frequent target of loss of heterozygosity (LOH) in cancer, and 8p LOH is commonly associated with a more aggressive tumor phenotype. The 8p11-12 region is a recurrent breakpoint area characterized by a sharp decrease in gains/amplifications and increase in allelic loss towards 8pter. However, the clustering of genomic aberrations in this region, even in the absence of proximal amplifications or distal LOH, suggests that the 8p11-12 region could play a pivotal role in oncogenesis.

**Results:**

Loss in the *FGFR1* and *ZNF703*-containing 8p11 region was seen in 25% of patients, correlated with lower mRNA expression levels and independently predicted poor survival, particularly in systemic treatment-naïve patients and even without adjacent 8p12 loss. Amplification of *FGFR1* at 8p11 and loss of *DUSP26* and *UNC5D*, located in the 8p12 breakpoint region, independently predicted worse event free survival. Gains in the 8p12 region encompassing *WRN*, *NRG1*, *DUSP26* and *UNC5D*, seen in 20-30% of patients, were associated with higher mRNA expression and independently predicted chemotherapy sensitivity. Losses at 8p12 independently predicted radiotherapy resistance.

**Material and methods:**

Multiplex ligation-dependent probe amplification was used to investigate copy number aberrations at 8p11-12 in 234 female breast cancers. Alterations were correlated with clinicopathologic characteristics, survival and response to therapy. Results were validated using public METABRIC data

**Conclusion:**

Allelic loss and amplification in the 8p11-12 breakpoint region predict poor survival and chemo- and radiotherapy response. Assessment of 8p11-12 gene copy number status seems to augment existing prognostic and predictive tools.

## INTRODUCTION

The short arm of chromosome 8 (8p) is a frequent target of loss of heterozygosity (LOH) in colorectal, hepatocellular, prostate, bladder, lung, ovarian as well as breast cancer [1–9]. Furthermore, LOH on 8p is more frequent in aggressive breast cancers with worse overall survival [10–14], in poorly differentiated and advanced stages of hepatocellular carcinoma [15], in advanced clinical stages of colorectal carcinomas [16], in bladder cancers of high grade and stage [17], and in prostate carcinomas with worse outcome [18, 19]. This suggests that (synergistic) loss or inactivation of tumor suppressor genes on 8p plays a role in the progression of multiple cancer types.

To establish molecular drivers of 8p loss, several studies have tried to pinpoint one or more regions of minimal deletion using either cell lines or human breast cancers [5, 12, 13, 20, 21]. The results have been inconsistent, with regions at 8p12, 8p12-21, 8p22 and 8p22-23.3 being reported. Recently, Cai *et al.* pinpointed a region commonly affected by 8p deletions using TCGA data, and subsequently examined the effect of a chromosome 8p 2-35 Mb targeted deletion, which was insufficient to transform MCF10A cells, but altered the fatty acid and ceramide metabolism leading to increased invasiveness and enhanced autophagy [14].

LOH of 8p is often but not always associated with amplification of the neighbouring 8p12-p11.23 region, containing *FGFR1* and *ZNF703*. Concomitant break and amplification are likely to result from the breakage-fusion-bridge mechanism [22]. However, the clustering of breakpoints, even in the absence of proximal amplifications or distal deletions, suggests that the 8p12 region is sensitive to breakages and could play a pivotal role in oncogenesis via inactivation of one or several tumor suppressor genes, and/or activation of one or more oncogenes. Adding to the complexity, the nature of specific driver genes and the type of copy number alteration in this breakpoint region may be context dependent. Here, we explore METABRIC data and confirm that 8p LOH is a frequent phenomenon in poor-prognosis breast cancer, with a sharp increase in the frequency of copy number loss between *ZNF703* (20% loss, 26% gain/amplification) and *WRN* (39% loss, 9% gain/amplification) at 8p12. Subsequently, we show in a large series of breast cancer patients that allelic loss and amplification in the 8p11-12 breakpoint region predict poor survival and chemoradiotherapy response. Regardless of why cancer cells select for amplifications in some settings and copy number loss in others, these results should increase consideration of the prognostic and predictive potential of copy number aberrations in this region.

## RESULTS

### METABRIC breakpoint identification

Supplementary Figure 1 depicts a heatmap of putative copy number calls on chromosome 8p observed in METABRIC (n=2173 informative cases). In 94% of cases, the copy number status of *FGFR1* and *ZNF703* was similar. However, a very strong putative breakpoint was observed in a more distal region between *ZNF703* (37553269 bp from pter) and *DUSP26* (33448851 bp from pter), where *DUSP26* was gained or amplified in 14% of cases (versus 26% of cases for *ZNF703*) and lost in 34% of cases (versus 20% for *ZNF703*). This region contains two other genes, *KCNU1* (36641842 bp from pter) and *UNC5D* (35092975 bp from pter). A putative breakpoint between *ZNF703* and *KCNU1* was observed in 155/2173 cases (7%) while putative breakpoints between *KCNU1* and *UNC5D* and between *UNC5D* and *DUSP26* were present in 204/2173 (9.4%) and 197/2173 (9.1%) cases. Breakpoints between *DUSP26* and neighbouring gene *RNF122* were however much less frequent (3/2173; 0.1%). Between *NRG1* and *WRN*, another weaker breakpoint was observed, with 36% and 39% loss, and 13% and 9% gain/amplification of *NRG1* and *WRN*, respectively.

### 8p11-12 copy number alterations by MLPA

Table 1 shows the frequency of allelic loss, copy number increase and high-level amplification observed in the investigated genes and genomic subregions and Table 2 shows the significant associations of these copy number alterations with pathological characteristics. Raw chromosome 8p MLPA and corresponding clinical/pathological data are provided in Supplementary Table 4. Overall, regardless of the genomic sub-region, copy number increase on the short arm of chromosome 8 was associated with high grade (p=0.037) and high mitotic activity (p=0.011). Copy number increase and high level amplification were most frequently observed in luminal B-like tumors (p=0.017 and p=0.003 respectively; 68% demonstrated a copy number increase at 8p). The presence of an allelic loss on the short arm of chromosome 8 was associated with ductal histology (p=0.027), PR negativity (p=0.031), high grade (p=0.008) and high mitotic activity (p=0.003). Allelic loss at 8p was most frequent in non-luminal A-like (p=0.003; 32% in luminal A-like) tumors and tended to be more prevalent in luminal B-like (p=0.053; 57%) and triple negative (p=0.065; 61%) tumors presenting at older age (p=0.057).

**Table 1:** Frequency of allelic loss, copy number increase (gain or amplification) and amplification (cut-off 2.0) by MLPA

Gene	Ensembl cytogenetic band	Distance from pter (Mbp)	Cut-off Loss	Cut-off Gain/Amp	% Loss	% Gain/Amp	% Amp
***WRN***	8p12	31.0	0.82	1.14	10	8	1
***NRG1***	8p12	31.6	0.81	1.17	9	10	1
***DUSP26***	8p12	33.6	0.80	1.31	16	10	1
***UNC5D***	8p12	35.2	0.78	1.43	13	12	3
***ZNF703***	8p11.23	37.7	0.71	1.37	9	31	15
***FGFR1***	8p11.23	38.4	0.82	1.16	12	19	7
***FNTA***	8p11.21	43.0	0.77	1.36	5	19	3
***PRKDC***	8q11.21	47.8	0.75	1.25	1	26	2
***8p12***	≥1 of 4 genes	-	-	-	36	31	4
***8p12 only***	≥1 of 4 genes	-	-	-	6	9	0
***transition***	≥1 of 3 genes	-	-	-	33	21	4
***transition only***	≥1 of 3	-	-	-	9	5	0
***8p11***	≥1 of 3 genes	-	-	-	26	42	16
***8p11 only***	≥1 of 3 genes	-	-	-	7	8	5
***8p11-12***	≥1 of 7 genes	-	-	-	44	52	17

Cut-offs for loss and gain/amplification were determined per gene, based on the minimum and maximum ratio values of normal breast tissue.

Transition = region including *NRG1*, *DUSP26* and *UNC5D.*

Only = without adjacent loss or gain/amp.

**Table 2:** The association of 8p copy number alterations with clinical/pathological characteristics

Loss vs Neutral	WRN	NRG1	DUSP26	UNC5D	8p12	ZNF703	FGFR1	FNTA	8p11	PRKDC (8q)
**Age**	>50 (0.043)		>50 (0.002)	>50 (0.047)	>50 (0.027)	>50 (0.048)				
**pN**										
**pT**										
**Grade**										
**Histological subtype**										
**MAI**									high (0.034)	
**ER**							**neg (0.036)**		**neg (0.015)**	
**PR**						**neg (0.038)**	**neg (0.017)**		**neg (0.011)**	
**HER2**										
**LumA-like**						**no (0.042)**			**no (p=0.013)**	
**LumB-like**					**yes (0.040)**					
**TN**									yes (0.044)	
**HER2-like**										
**Gain/amp vs Neutral**	**WRN**	**NRG1**	**DUSP26**	**UNC5D**	**8p12**	**ZNF703**	**FGFR1**	**FNTA**	**8p11**	**PRKDC (8q)**
**Age**										
**pN**										
**pT**	<=2cm (0.036)									
**Grade**										
**Histological subtype**										
**MAI**							high (0.048)		high (0.039)^*^	high (0.027)^*^
**ER**				**pos (0.033)**		pos (0.007)^*^				pos (0.015)
**PR**								pos (0.011)		pos (0.005)
**HER2**					neg (0.014)					
**LumA-like**										
**LumB-like**				**yes (0.018)**		yes (0.026)^*^	**yes (0.002)**		yes (0.016)^*^	**yes (0.008)**
**TN**						**no (0.036)**				
**HER2-like**										

Bold indicates a similar association in METABRIC. Gain/amp values with asterisk (^*^) indicate associations also seen at MLPA cut-off 2.0 (high-level amplification).

Copy number increase of at least 1 of the 4 genes in the 8p12 region (*WRN*, *NRG1*, *DUSP26* and/or *UNC5D*) was associated with HER2 negativity (p=0.014). Copy number increase, especially high-level amplification of at least 1 of the 3 genes in the 8p11 region (*ZNF703*, *FGFR1* and/or *FNTA*) was predominantly seen in luminal B-like tumors (p=0.016 and p=0.005, respectively) and tumors with high mitotic counts (p=0.039 and p=0.016, respectively). Allelic loss of at least 1 gene in the 8p12 region was more prevalent in luminal B-like tumors (p=0.040) and tumors diagnosed at older age (p=0.027). Allelic loss of the 8p11 genomic subregion was associated with ER and PR negativity (p=0.015 and p=0.011, respectively), high mitotic activity (p=0.034), a non-luminal A-like subtype (p=0.013) and triple negativity (p=0.013; 45% demonstrated allelic loss at the 8p11 region). Supplementary Figure 2 depicts the frequencies of 8p regional copy number alterations in each molecular breast cancer subtype.

### FGFR1 MLPA versus FISH

Paired MLPA-FISH data were available for 179 tumors. As illustrated in Supplementary Figure 3, 17/20 (85%) cases with loss by MLPA showed a loss by FISH as well, and 27/28 (96%) cases showing gain or amplification by MLPA were also gained/amplified by FISH. 114/131 (87%) cases with no alterations by MLPA showed normal FISH copy numbers. The Pearson correlation between MLPA and FISH was strong (r=0.855, p<0.05). In 50% of cases, CEP8 was increased when *FGFR1* copy number was increased and in 33% of cases, CEP8 demonstrated copy number loss when *FGFR1* showed loss, suggesting that 8p11 loss or gain/amp is not always the result of chromosome 8 polysomy or monosomy. All cases with CEP8 loss also showed *FGFR1* loss, and the majority (67%) of CEP8 gained cases also demonstrated *FGFR1* copy number increase.

### Chromosome 8p copy number alterations predict worse prognosis

**Figure 1:**
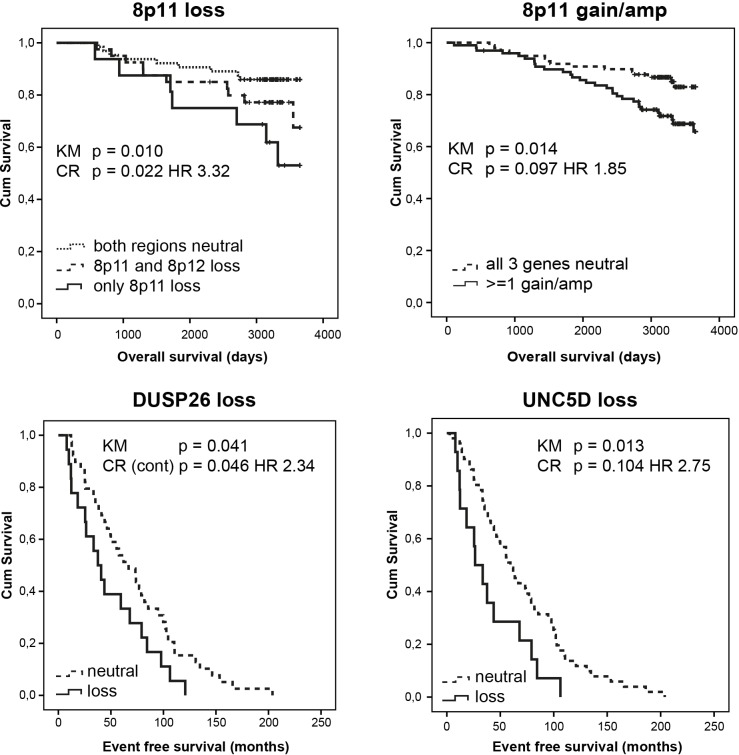
Allelic loss and gain/amplification at 8p11 independently predict poor overall survival in a set of 234 female breast tumors analysed by MLPA *DUSP26* and *UNC5D* copy number loss independently predict worse event free survival. KM = Kaplan Meier analysis; CR = Cox Regression analysis; HR = hazard ratio.

Table 3 summarizes unadjusted log-rank p-values and adjusted hazard ratios (HR) of 8p genes and (sub)regions significantly associated with overall (OS) and event free (EFS) survival. Copy number increase (gain or amplification) of *DUSP26*, and allelic loss of *DUSP26* and *UNC5D* individually predicted OS. Overall, the presence of ≥1 gain/amplification or ≥1 loss within the 8p11-12 region was indicative of a worse OS in univariable analysis. As for the genomic subregions, gain/amplification or loss at 8p11, even without adjacent 8p12 copy number aberrations (Figure 1), predicted poor OS. After correction for age, stage, intrinsic subtype, tumor size, ER/PR/HER2 status, grade, MAI, and LN status, loss within the 8p11 region, especially without adjacent 8p12 loss, was an independent prognostic variable for OS (p=0.022; HR 3.3). Gain/amplification in the 8p11 region also independently predicted poor OS. The presence of at least one high-level amplification at 8p was indicative for worse EFS in multivariable analysis (p=0.038). Only in ER-positive tumors, allelic loss at the 8p12 subregion was associated with worse EFS (p=0.041). Even in the generally less aggressive luminal A-like tumors, 8p12 loss or gain/amplification predicted worse EFS (p=0.045 and p=0.004, respectively). Individually, *DUSP26* and *UNC5D* copy number loss (Figure 1) and *ZNF703* and *FGFR1* amplification independently predicted worse EFS.

**Table 3:** Unadjusted log-rank p-values and adjusted hazard ratios (HR) of 8p genes and (sub)regions significantly associated with overall (OS) and event free (EFS) survival

OS	*Univariate*	Cox proportional hazards^*^		
**Gain/amplification**		***p-value***	***HR***	***95% CI***
*DUSP26*	0.017	0.012 (cat)/0.043 (avg)	2.913/2.891	1.271-6.678/1.034-8.086
8p11	0.014	0.097	1.848	0.895-3.816
8p11 only	0.033	-	-	-
8p11-12	0.043	-	-	-
**Amplification**		***p-value***	***HR***	***95% CI***
*ZNF703*	-	-	-	-
*FGFR1*	-	-	-	-
8p11-12	-	-	-	-
**Loss**		***p-value***	***HR***	***95% CI***
*DUSP26*	-	-	-	-
*UNC5D*	-	-	-	-
8p11	0.012	0.065	2.092	0.955-4.585
8p11 only	0.010	0.022	3.323	1.189-9.284
8p11-12	0.017	0.084	2.045	0.908-4.606
**EFS**	***Univariate***	Cox proportional hazards^**^		
**Gain/amplification**		***p-value***	***HR***	***95% CI***
*DUSP26*	-	-	-	-
8p11	-	-	-	-
8p11 only	-	-	-	-
8p11-12	-	-	-	-
**Amplification**		***p-value***	***HR***	***95% CI***
*ZNF703*	-	0.036	3.198	1.081-9.462
*FGFR1*	-	0.010	10.809	1.767-66.118
8p11-12	-	0.038	4.721	1.090-20.440
**Loss**		***p-value***	***HR***	***95% CI***
*DUSP26*	0.041	0.046 (cat)/0.089 (cont)	2.340/0.388	1.017-5.383/0.130-1.157
*UNC5D*	0.013	0.104 (cont)	0.364	0.108-1.232
8p11	-	-	-	-
8p11 only	-	-	-	-
8p11-12	-	-	-	-

Comparison versus neutral copy number.

^*^adjusted for age (cont), stage, intrinsic subtype, tumor size (cont), ER/PR, Grade, MAI (cont), LN status.

^**^adjusted for age (cont), intrinsic subtype, tumor size (cont), ER/PR/HER2, Grade, MAI, LN status.

cont = as continuous variable (raw MLPA ratio); cat = as categorical variable (cut-off based on normal tissue).

### Chromosome 8p copy number alterations predict response to therapy

Supplementary Table 5 summarizes adjusted hazard ratios (HR) of 8p genes and (sub)regions significantly associated with EFS in different treatment categories. In CT-naïve patients, Cox-regression analysis indicated a better EFS if 8p11 or 8p11-12 losses (p=0.035 and p=0.02, respectively) or gains/amplifications (p=0.004 and p=0.004, respectively) were present, and in case of *NRG1* loss (p=0.042). In CT-treated patients, however, multivariable analysis indicated a worse EFS if 8p12 or 8p11-12 losses were present (p=0.002 and p=0.082, respectively), and better EFS when 8p12 gains were present (p=0.009), suggesting an association between 8p copy number alterations and CT response. Also individually, loss of *WRN* (p=0.001), *NRG1* (p<0.001), *DUSP26* (p<0.001), *UNC5D* (p<0.001), *ZNF703* (p<0.001) or *FNTA* (p=0.001) and copy number increase of *ZNF703* (p=0.052 gain/amp and p=0.011 amp), *FGFR1* (p=0.038 gain/amp and p=0.003 amp) and *FNTA* (p=0.046 amp) independently predicted worse EFS in patients receiving CT.

In HT-naïve patients, multivariable analysis indicated worse EFS when tumors showed 8p11 (p=0.037), 8p12 (p=0.053) or 8p11-12 (p=0.026) loss, and better EFS for tumors with 8p12 gains (p=0.037). Individually, *WRN* (p=0.011), *NRG1* (p=0.006), *DUSP26* (p=0.001) and *UNC5D* (p=0.001) copy number decrease independently predicted worse EFS in HT-naïve patients. In patients receiving HT, these associations were no longer apparent but *ZNF703*, *DUSP26* and 8p11 amplification independently predicted better EFS. In patients receiving RT, 8p12 loss (p=0.034) and *DUSP26* copy number loss (p=0.053) independently predicted worse EFS.

### METABRIC confirms prognostic and predictive value of 8p copy number alterations

Supplementary Table 6 shows METABRIC copy number variation frequencies of all chromosome 8 genes interrogated by MLPA. Supplementary Table 7 shows the association between chromosome 8 copy number variations by METABRIC and ER/PR/HER2 status, surrogate intrinsic subtype, tumor size, grade, stage and mutation load. In METABRIC tumors, allelic loss of at least one of the interrogated genes was significantly correlated with ER negativity (p=0.000018; 57% of ER- tumors), PR negativity (p<0.0000001; 57%), HER2 positivity (p<0.0000001; 69%) and a non-luminal A-like (p<0.0000001; 60%), luminal B-like (p<0.0000001; 61%) or HER2-driven (p<0.0000001; 67%) surrogate intrinsic subtype. Tumors were larger (p=0.001; 51%) and of higher stage (p=0.007; 61%) and grade (p<0.0000001; 62%) compared to copy number neutral tumors. There was no association with age. In the METABRIC dataset, copy number increase of at least one of the 7 interrogated genes was significantly associated with older age (p=0.048) and, similar to copy number losses in this region, with large tumor size (p=0.014), high grade (p<0.0000001) and stage (p=0.004), PR negativity (p=0.0001), HER2 positivity (p=0.005), non-luminal A-like (p<0.0000001), luminal B-like (p<0.0000001) and HER2-driven (p=0.014) surrogate intrinsic subtype. Overall, upon comparison of our data with METABRIC, we were able to confirm the positive association between chromosome 8p11-12 loss, PR negativity and high grade, and the negative association between these losses (particularly *ZNF703* loss) and the luminal A subtype. Furthermore, METABRIC confirmed the association between 8p11-12 gain, high grade and the luminal B-like subtype.

**Figure 2:**
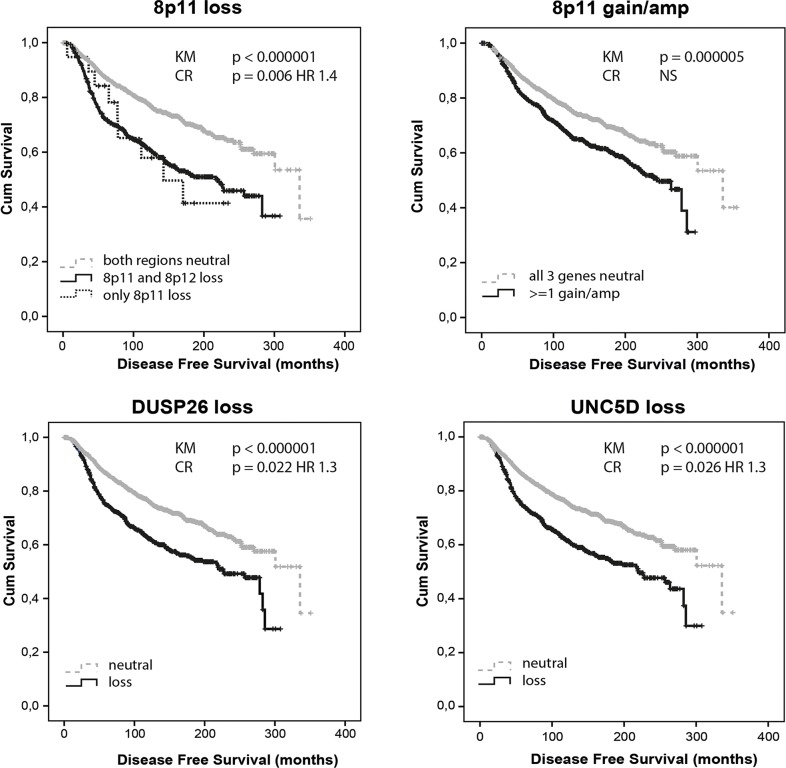
Allelic loss and gain/amplification at 8p11 predict poor disease free survival in METABRIC *DUSP26* and *UNC5D* copy number loss independently predict worse disease free survival. KM = Kaplan Meier analysis; CR = Cox Regression analysis.

In METABRIC, copy number loss at 8p11-12, 8p11, 8p12 and 8p LOH were correlated with worse disease free survival (DFS; all p<0.000001). Also, patients showing 8p11 loss without adjacent 8p12 loss showed a significantly worse DFS compared to patients where both regions were copy number neutral (p=0.028; Figure 2). Individually, the loss of all interrogated genes correlated with worse DFS (all p<0.000001 except *FNTA* p=0.000001 and *PRKDC* p=0.0002). Amplification of 8p11-12, 8p11 and to a lesser extent 8p12 correlated with worse DFS too (p=0.00001, p=0.00001 and p=0.011, respectively). Individually, *DUSP26*, *UNC5D*, *ZNF703*, *FGFR1*, *FNTA* and *PRKDC* amplifications predicted worse DFS (p=0.021, p=0.001, p=0.003, p=0.00006, p=0.002 and p=0.001, respectively). The correlation between copy number loss or gain/amp and worse survival was most prominent in ER positive HER2 negative tumors with high proliferation. Also within PAM50-classified luminal A tumors, 8p12 loss (p=0.007), 8p11 loss (p=0.012) and 8p11-12 loss (p=0.005) predicted worse DFS. After correction for age, tumor size, stage and grade, ER/PR/HER2 status and 3-gene classifier subtype, the loss of *WRN* (p=0.053; HR 1.2), *DUSP26* (p=0.022; HR 1.3), *UNC5D* (p=0.026; HR 1.3), *ZNF703* (p=0.026; HR 1.3) and *FGFR1* (p=0.002; HR 1.5) still predicted worse DFS. Of these 5 genes entered simultaneously in the model, *DUSP26* (p=0.014; HR 1.37; 95% CI 1.07-1.77) remained as best DFS predictor. Overall a loss within the 8p11 (p=0.006; HR 1.40; 95% CI 1.1-1.77) but not the 8p12 subregion independently predicted worse survival. Amplification at 8p11-12 (p=0.047; HR 1.30; 95% CI 1.00-1.69), 8p11 (p=0.033 HR 1.32; 95% CI 1.02-1.70) and *FGFR1* (p=0.007 HR 1.47; 95% CI 1.11-1.94) also remained in the model when corrected for other prognostic variables. As compared to our dataset, METABRIC thus confirmed 8p11 loss, even without adjacent 8p12 loss, *DUSP26* loss, *UNC5D* loss, *FGFR1* amplification and amplification at 8p11-12 as independent predictors of worse survival.

**Figure 3:**
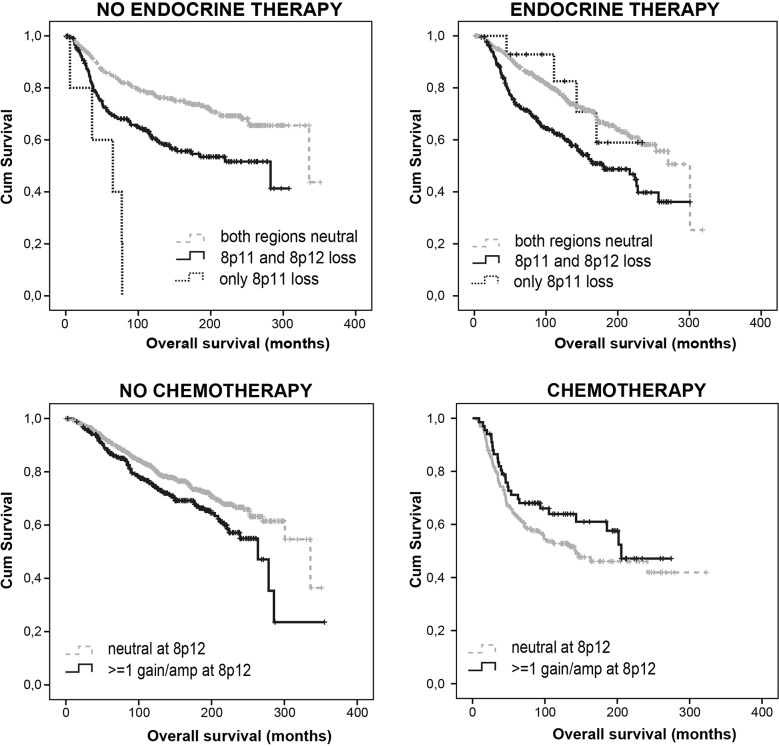
Loss at 8p11 predicts poor prognosis, particularly in endocrine treatment naïve patients, while gain at 8p12 predicts chemotherapy sensitivity Figure based on METABRIC data.

Supplementary Table 8 summarizes adjusted HR of 8p genes and (sub) regions significantly associated with DFS in different treatment categories of the METABRIC dataset. 8p12 gains independently predicted better DFS in CT-treated patients (p=0.040; HR 0.58; 95% CI 0.34-0.98) but not in CT-naïve patients, suggesting a correlation with chemotherapy sensitivity. *FNTA* amplifications predicted worse DFS in CT-treated patients (p=0.047) but not in CT-naïve patients, suggesting a correlation with CT resistance. *FGFR1* losses independently predicted worse DFS in RT-treated patients (p=0.033; HR 1.39; 95% CI 1.03-1.88) but not in RT-naïve patients, suggesting a correlation with radiotherapy resistance. In addition, a loss of at least 2 genes of *NRG1*,*DUSP26* and *UNC5D* predicted RT resistance as well (p=0.02; HR 1.35; 95% CI 1.05-1.73). *WRN* loss (p=0.018; HR 1.40), *UNC5D* loss (p=0.017; HR 1.43), *ZNF703* loss (p=0.023; HR 1.47), *FGFR1* loss (p=0.002; HR 1.70) or amplification (p=0.083; HR 1.36), *PRKDC* loss (p=0.045; HR 1.74), a loss of at least 2 genes of *NRG1*, *DUSP26* and *UNC5D* (p=0.016; HR 1.38; 95% CI 1.06-1.80) and LOH at 8p (p=0.021; HR 1.36; 95% CI 1.05-1.76) predicted worse DFS in HT-treated patients but not in HT-naïve patients, suggesting a correlation with HT resistance. Loss at 8p11 and especially loss at 8p11 without adjacent 8p12 loss were independent predictors of poor DFS in HT-naïve patients only (p=0.035; HR 1.481 and p= 0.000; HR 7.123, respectively). Amplification at 8p11 was also independently indicative of worse DFS in CT/HT/RT treatment-naïve patients. As compared with our dataset, METABRIC confirmed 8p12 gain as a predictor of CT sensitivity (Figure 3), *FNTA* amplification as a predictor of CT resistance, (focal) 8p11 loss/amplification as a strong independent predictor of poor prognosis in HT-naïve patients, and LOH in the *NRG1*/*DUSP26*/*UNC5D* breakpoint region as a predictor of RT resistance.

### Correlation between copy number loss, mRNA expression and survival in METABRIC

**Figure 4:**
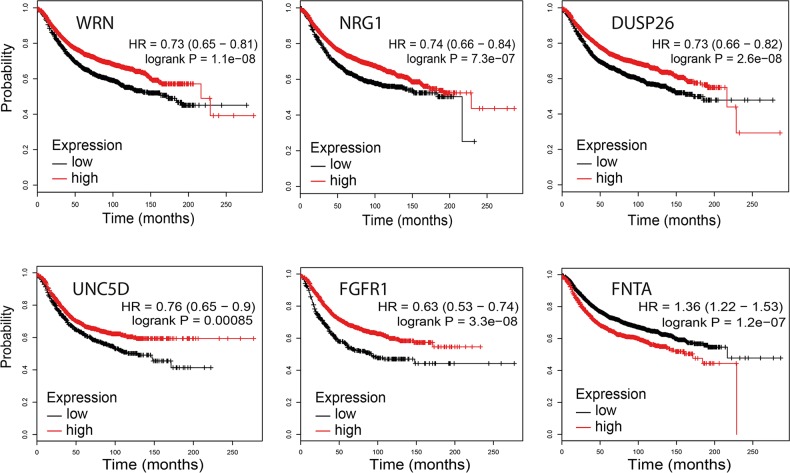
Association between WRN, NRG1, DUSP26, UNC5D, FGFR1 and FNTA mRNA expression levels and relapse free survival (KMplotter)

*WRN* (p=1E-13), *ZNF703* (p=2E-9), *FGFR1* (p=1E-10), *FNTA* (p=1E-13) and *PRKDC* (p=1E-13) demonstrated a significant correlation between copy number loss and mRNA expression downregulation (Supplementary Figure 4). All interrogated genes demonstrated a significant correlation between copy number gain and mRNA upregulation (*WRN* p=1E-13; *NRG1* p=0.016; *DUSP26* p=0.004; *UNC5D* p=0.042; *ZNF703* p=1E-13; *FGFR1* p=1E-13; *FNTA* p=1E-13 and *PRKDC* p=1E-13). mRNA expression of *NRG1*, *DUSP26* and *UNC5D* was low (median Z score < 0) regardless of the copy number status, suggesting that alternative mechanisms such as DNA promoter hypermethylation may play a more important role here. The Kaplan Meier Plotter (http://kmplot.com/analysis/) was used to determine the relationship between mRNA expression and relapse free survival in 3951 breast cancer samples [31]. For WRN (p=1.1E-08; HR 0.73, 95% CI 0.65-0.81), NRG1 (p=7.3E-07; HR 0.74, 95% CI 0.66-0.84), DUSP26 (p=2.6E-08; HR 0.73, 95% CI 0.66-0.82), UNC5D (p=0.00085; HR 0.76, 95% CI 0.65-0.9) and FGFR1 (p=3.3E-8; HR 0.63, 95% CI 0.53-0.74), mRNA downregulation predicted worse relapse free survival (Figure 4). In contrast, for FNTA (p=1.2E-07; HR 1.36; 95% CI 1.22-1.53) and PRKDC (p=4.9E-08; HR 1.38; 95% CI 1.23-1.56), a mRNA upregulation predicted worse survival. For ZNF703, no data were available.

## DISCUSSION

The 8p11-12 genomic region is characterized by a strong 4 MB putative breakpoint region between *ZNF703* (37553269 bp from pter) and *DUSP26* (33448851 bp from pter). This study investigated the clinical relevance of copy number alterations in the 8p11-12 breakpoint region in breast cancer. We have shown and confirmed by METABRIC that both gain/amplification and loss in this region correlate with a more aggressive tumor phenotype. The presence of at least one high-level amplification at 8p11-12, in particular *FGFR1* amplification, was an independent predictor of poor survival in both datasets. Interestingly, although it has long been assumed that copy number loss at the *FGFR1*-containing 8p11 region is a bystander effect of deletions occurring more distal on chromosome 8p12, 8p11 loss was an independent predictor of poor OS in both datasets, even without adjacent 8p12 loss. Although copy number increase at the 8p11 region was independently correlated with worse OS in our dataset, this association was secondary to other tumor characteristics in METABRIC. Additionally, loss of *DUSP26* and *UNC5D*, located in the 8p12 breakpoint region, independently predicted worse survival. Interestingly, in both datasets, allelic loss at 8p12 was associated with worse EFS or DFS within the luminal A(-like) subtype, suggesting a prognostic role of 8p copy number alterations within this subtype as well. In glioblastoma, *DUSP26* mRNA expression is downregulated and plays a role in intracellular transport and cell-cell adhesion [23]. In epithelial cells, *DUSP26* negatively affects proliferation [24] and interacts with/is activated by adenylate kinase 2 (AK2). This complex is able to dephosphorylate fas-associated protein with death domain (FADD) to downregulate cell growth [25]. On the other hand, *DUSP26* seems to have oncogenic as well as tumor suppressor characteristics. In thyroid cancer, for example, *DUSP26* is amplified and promotes cell growth by inhibiting the p38 MAPK activity [26]. *UNC5D*, a netrin receptor involved in apoptosis, was described as tumor suppressor gene in renal cell carcinoma [27] and bladder cancer [28], and was shown to reside in a genetic locus predisposing to colon carcinoma in mice [29].

Gains/amplifications in the 8p12 region encompassing *WRN*, *NRG1*, *DUSP26* and *UNC5D*, were seen in 20-30% of patients, were indicative of higher mRNA expression levels and independently predicted CT sensitivity. Losses at 8p12 independently predicted RT resistance but for most genes located in this region, mRNA expression levels were low regardless of copy number levels suggesting alternative regulatory mechanisms such as promoter hypermethylation. In line with these observations, DUSP26 functions as a p53 phosphatase [30] and UNC5D as a p53 target gene [31]. Both genes may therefore modulate chemo- and radiotherapy response. Overexpression of DUSP26 was shown to sensitize PC12 cells to cisplatin-induced apoptosis [32]. Furthermore, *DUSP26* mRNA expression was enhanced by treatment of ovarian cancer cell lines with 5-aza-2-deoxycytidine (demethylation) and trichostatin A (HDAC inhibitor) [24]. In bladder cancer and neuroblastoma cell lines, *UNC5D* knockdown decreased sensitivity to cisplatin, mediated through E2F1, p53 and DAPK [28, 31, 33]. In addition, histone deacetylase and methylation inhibitors were able to restore UNC5D expression [27, 33]. Our study did observe an association between *DUSP26* and *UNC5D* loss and CT resistance, but we were not able to confirm this finding in METABRIC. Also located at 8p12 is *NRG1*. The neuregulin-1 gene has been proposed both as a candidate oncogene [34] and as a candidate tumor suppressor gene [35]. It encodes ligands that bind the ERBB/HER family of receptors (ERBB3/4) [35]. Although the NRG1-encoded proteins are usually thought of as mitogens, they can also be pro-apoptotic upon forced expression [36]. Elevated NRG1 expression in tumors lacking HER2 amplification is strongly associated with lapatinib sensitivity *in vitro* [37]. Chua et al. reported significant *NRG1* methylation in breast cancer cell lines and demonstrated that, after treatment with aza-deoxycitidine, the transcription of *NRG1* was activated. In addition, *NRG1* was identified as one of 15 breast cancer anti-estrogen resistance genes [38]. The fourth gene interrogated at 8p12 was WRN, a member of the RecQ family of DNA helicases that possesses a 3′-5′exonuclease activity. The main functions of WRN are in DNA repair and in telomere maintenance. WRN expression may play a role in chemoradiotherapy response by limiting DNA damage and replicative stress and thus preventing senescence [39–41]. Colorectal tumors lacking WRN have been shown to be more sensitive to topoisomerase I inhibitors and DNA-damaging agents, as conversion of treatment-induced single strand breaks into double strand breaks occurs at a high frequency in absence of WRN [42, 43].

Loss in the 8p11 region (*ZNF703*,*FGFR1* and *FNTA*) was seen in about 25% of patients, correlated with lower mRNA expression levels and independently predicted poor survival, particularly in treatment-naïve patients. Although amplifications of *FGFR1* and *ZNF703* have been extensively investigated in line with their roles as a proto-oncogenes and predictors of endocrine therapy resistance [44–50], allelic loss of these genes has only scarcely been described. Copy number loss at 8p11, encompassing *FGFR1* and *ZNF703*, was associated with ER/PR negativity and a non-luminal A-like phenotype. Our data pointed towards an association with triple negativity while METABRIC suggested an association with a HER2-driven or luminal B-like phenotype.

Copy number increase in the 8p11 region correlated with higher mRNA expression levels and was an independent predictor of worse OS in our dataset. Nevertheless, in METABRIC, the association with survival was secondary to other tumor/patient characteristics. Amplification of *FNTA* was however associated with CT resistance in both datasets. Disagreement between our and METABRIC's findings might be related to differences in cohort characteristics. For example, compared to our cohort, METABRIC contains generally older patients, fewer T1 and more poorly differentiated tumors, and more CT/RT-naïve patients. FISH analysis demonstrated that the majority of *FGFR1* copy number alterations at 8p11 were not a consequence of complete polysomy or monosomy of chromosome 8 but probably the result of focal deletions/amplifications. This is in line with MLPA data showing only few patients with complete 8p12 and 8p11 loss or amplification.

Why cancer cells select for amplifications in some settings and copy number loss in others, is likely context dependent. Many of the interrogated genes have been described as oncogenes as well as tumor suppressor genes, with opposite roles in different cancer types and even different roles during tumor progression. This intriguing contrast complicates development of new treatment strategies. Future studies are thus needed to investigate whether gain or loss at 8p11-12 justifies heavier or adjusted treatment schedules, or may be associated with other genetic or epigenetic changes that are directly targetable.

In conclusion, we have demonstrated that gain and amplification but also loss in the 8p11-12 region correlate with a more aggressive breast cancer phenotype. Assessment of 8p11-12 gene copy number status seems to augment existing prognostic and predictive tools, and deserves to be wider studied in prospective clinical studies.

## MATERIALS AND METHODS

### Tissue selection and DNA isolation

244 formalin fixed paraffin embedded female primary breast cancer specimens were obtained from the archives of the Department of Pathology of the University Medical Center Utrecht, The Netherlands. For all patients, clinical follow-up (mean/median follow-up of approximately 8 years) was available from the Netherlands Cancer Registry (NCR) and pathological characteristics were extracted from the nationwide network and registry of histo- and cytopathology in the Netherlands (PALGA). Use of anonymous or coded ‘left over’ material for scientific purposes does not require informed consent according to Dutch legislation (Medical Research Involving Human Subjects Act) and therefore obtains exempt from our institutional medical ethical review board [51].

Prior to DNA extraction, hematoxylin-eosin stained slides were reviewed by an experienced pathologist (PvD) to confirm the presence of malignancy. All samples were estimated to have a tumor percentage of at least 60%. Areas with lymphocytic infiltrate or ductal carcinoma *in situ* were avoided. Tumor tissue was scraped off from the marked tumor area on four 4 μm thick paraffin sections, and incubated overnight in proteinase K (10 mg/ml; Roche, Almere, The Netherlands) at 56°C followed by heat inactivation, centrifugation and recovery of the supernatant.

### Multiplex ligation-dependent probe amplification

MLPA was performed according to the manufacturer's (MRC Holland, Amsterdam, The Netherlands) instructions using the SALSA MLPA Breast Tumor probemix P078-C1 [52–54] for *FGFR1*, and a custom synthetic MLPA kit containing 11 probes against *WRN* (2 probes), *NRG1* (2 probes), *DUSP26* (2 probes), *UNC5D* (2 probes), *ZNF703* (1 probe), *FNTA* (1 probe) and *PRKDC* (1 probe; on 8q). For the latter, probes were mixed with the SALSA P200-A1 MLPA reference probemix, containing 11 reference probes for normalisation. Supplementary Table 1 shows the locations and partial sequences of the chromosome 8 probes in both probemixes. For genes with more than one probe present, the arithmetic mean of all the probe peaks of this gene in duplicate was calculated. Cut-offs for loss and copy number increase (gain or amplification) were determined per gene, based on the minimum and maximum ratio values of normal breast tissue taken along each MLPA run (Table 1). Amplification was defined as an MLPA ratio larger than 2.0. PCR was performed on 244 tumors, of which 5 samples were excluded from the custom MLPA kit, (n=239) and 10 samples from the P078-C1 MLPA kit based on quality assessment (n=234). Paired data were available for 234 tumors. Supplementary Table 2 shows basic clinical and pathological characteristics of the patients and primary tumors studied. All data generated or analysed during this study are included in this published article and its supplementary information files.

### METABRIC data extraction

METABRIC clinical data and copy number alteration (CNA) and mRNA Expression z-Scores (U133 microarray) from chromosome 8 were downloaded via The cBioPortal for Cancer Genomics [55, 56]. Putative gene copy number calls from DNA copy were used to extract amplification and deletion status and to create a chromosome 8 heatmap. Supplementary Table 3 shows basic clinical and pathological characteristics of METABRIC patients and primary tumors studied. Mean and median follow-up was 10.4 years and 9.7 years, respectively.

### Fluorescence in situ hybridisation

FISH was performed on TMA sections (containing a subset of 217 tumors) using a three-colour FGFR1 Breakapart/Amplification probe (Cytocell, LPS 018). A detailed description of the FISH procedure can be found in Supplementary Methods. Based on comparison with MLPA, we defined an *FGFR1* copy number lower than 1.34 as loss, and higher than 3.17 as gain or amplification.

### Statistical analysis

Breast tumors were classified into Luminal A (ER+ and/or PR+, HER2-, mitotic activity index (MAI)≤14), luminal B (ER+ and/or PR+ and (HER2+ or MAI>14)), HER2 driven (ER-, PR-, HER2+) and triple negative (ER-, PR-, HER2-) subtypes. The MAI was determined by examining stained slides of tumor and counting the number of visible mitotic figures in the area containing the highest density of mitotic figures. The total number of mitotic figures counted in an area of 2 mm2 is the MAI. The variables ER (> 10%), PR (>10%), HER2 (3+), MAI (>14), tumor size (>T1), lymph node status (positive vs. negative), age (>50), and grade using the Nottingham Histologic Score system (the Elston-Ellis modification of Scarff-Bloom-Richardson grading system, 3 vs 1/2) were categorized for Chi Square statistics.

MLPA gene copy number data were grouped as loss, neutral, gain/amplification or amplification. In addition, four genomic sub-regions were investigated for the presence of copy number alterations and their associations with classical pathological characteristics: the 8p12 region (*WRN*, *NRG1*, *DUSP26* and *UNC5D*), the 8p11 region (*ZNF703*, *FGFR1* and *FNTA*) and the combination of both these regions (defined as 8p11-12).

The Mann-Whitney test was used to evaluate the association between copy number alterations (versus neutral copy number) and mRNA expression z-scores by METABRIC. Overall survival (OS), event free survival (EFS; event = recurrence or metastasis), and disease free survival (DFS; METABRIC) curves were constructed using the Kaplan–Meier method and the log-rank test was used to test for significance. Multivariate survival analysis was performed using a backward Cox proportional hazards model. Characteristics with a p<.10 in univariate analysis and potential confounders were included.

All statistical analyses were conducted with IBM SPSS 21 statistical software, regarding two-sided p-values below 0.05 as significant. For survival, at least 5 non-censored events in each category were required for consideration.

## SUPPLEMENTARY MATERIALS FIGURES AND TABLES
















