# Supplementary material for: Copy number changes at 8p11-12 predict adverse clinical outcome and chemo- and radiotherapy response in breast cancer
Source: Oncotarget. 2018 Mar 30;9(24):17078–92. doi: 10.18632/oncotarget.24904 (PMC5908307; doi:10.18632/oncotarget.24904)
Supplement: Supplementary file 1 [file oncotarget-09-17078-s001.pdf]

Copy number changes at 8p11-12 predict adverse clinical outcome and chemo- and radiotherapy response in breast cancer

SUPPLEMENTARY MATERIALS

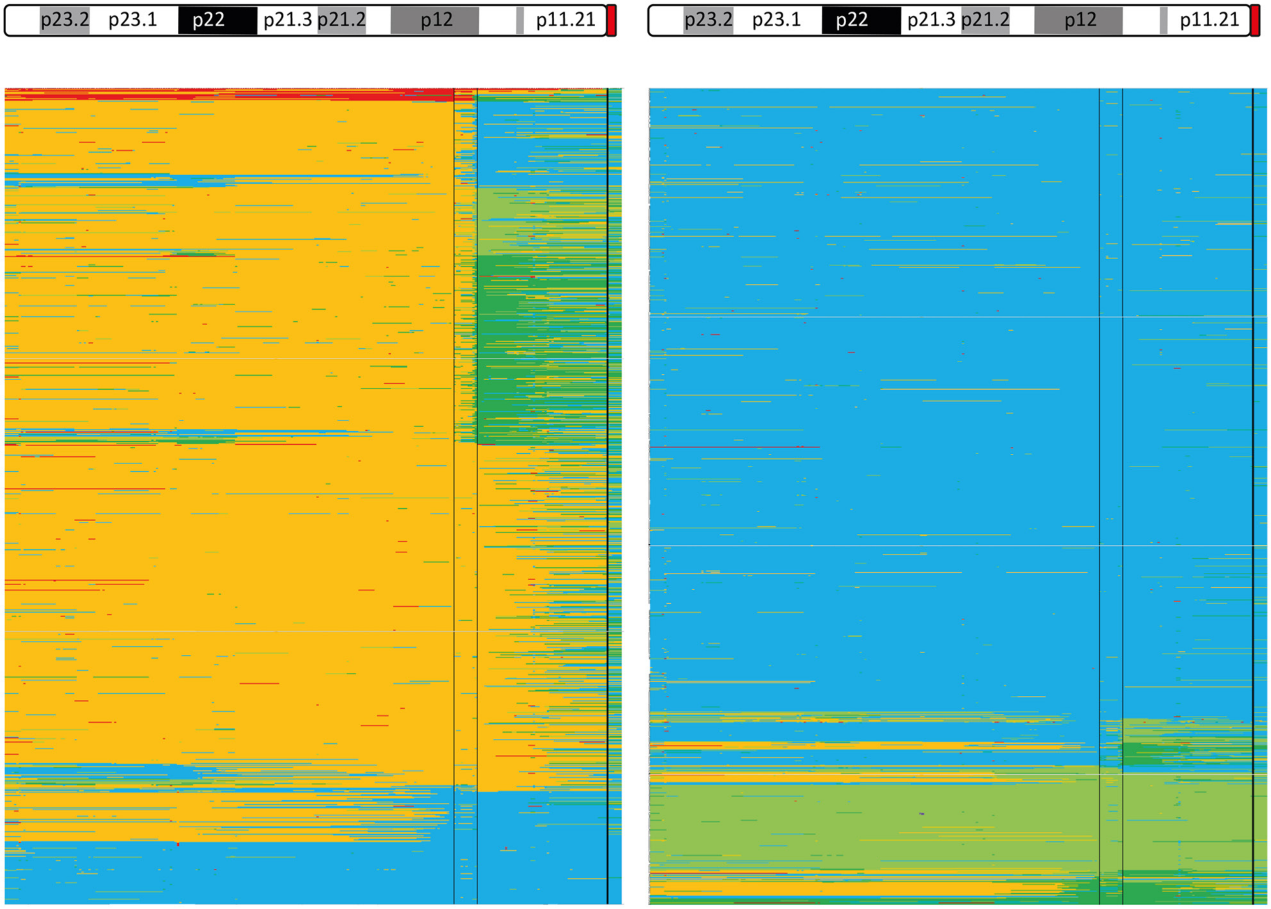

Supplementary Figure 1: Heatmap of putative copy number calls on chromosome 8p observed in METABRIC (n=2173 informative cases; each row represents one patient). Depicted are homozygous (red) and hemizygous deletion (orange), copy number neutral (blue), gain (light green) and high-level amplification (dark green). Regular vertical lines indicate the breakpoint region between *ZNF703* and *WRN*.

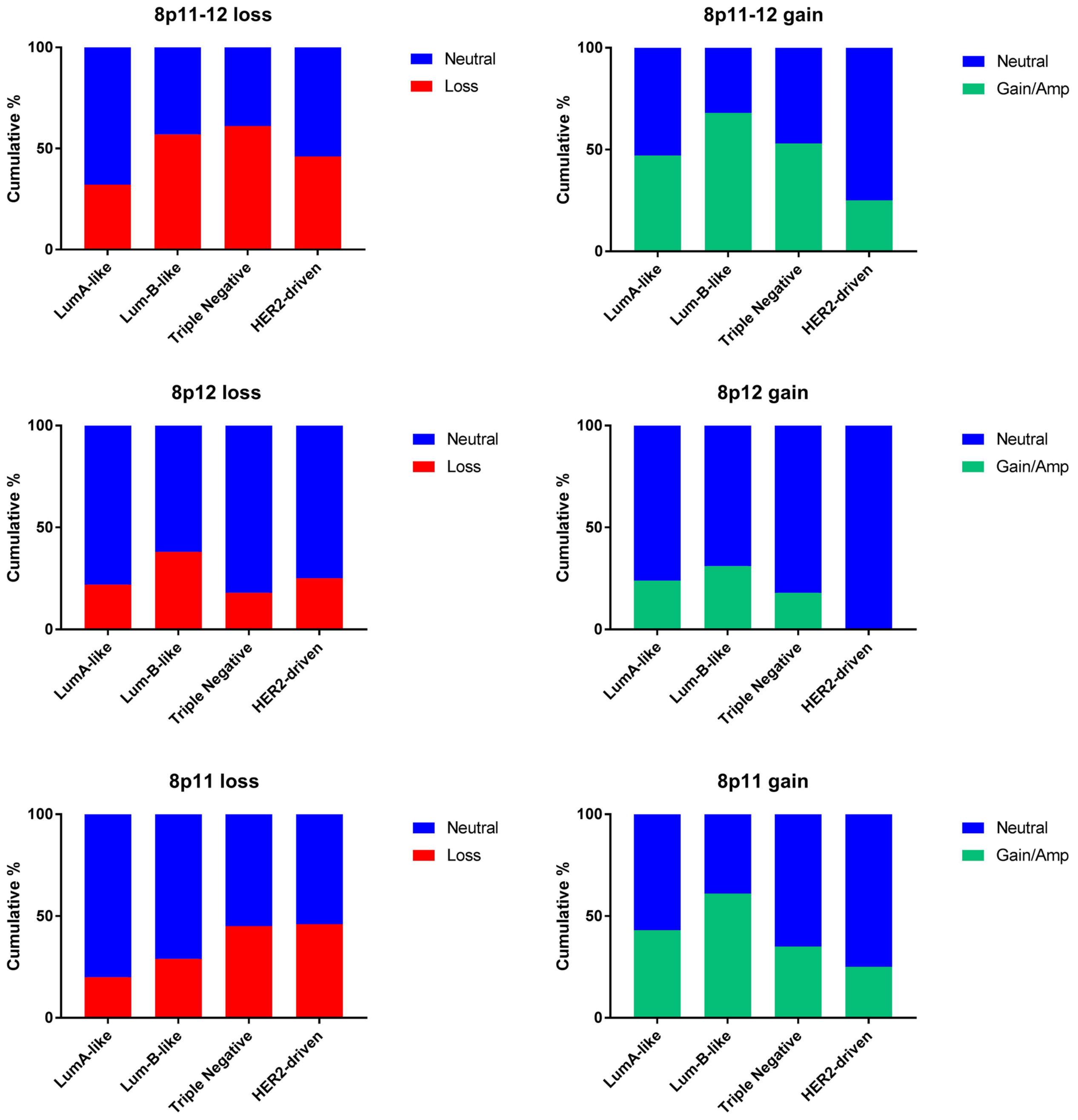

Supplementary Figure 2: Association between 8p11-12 copy number alterations and surrogate intrinsic molecular subtype.

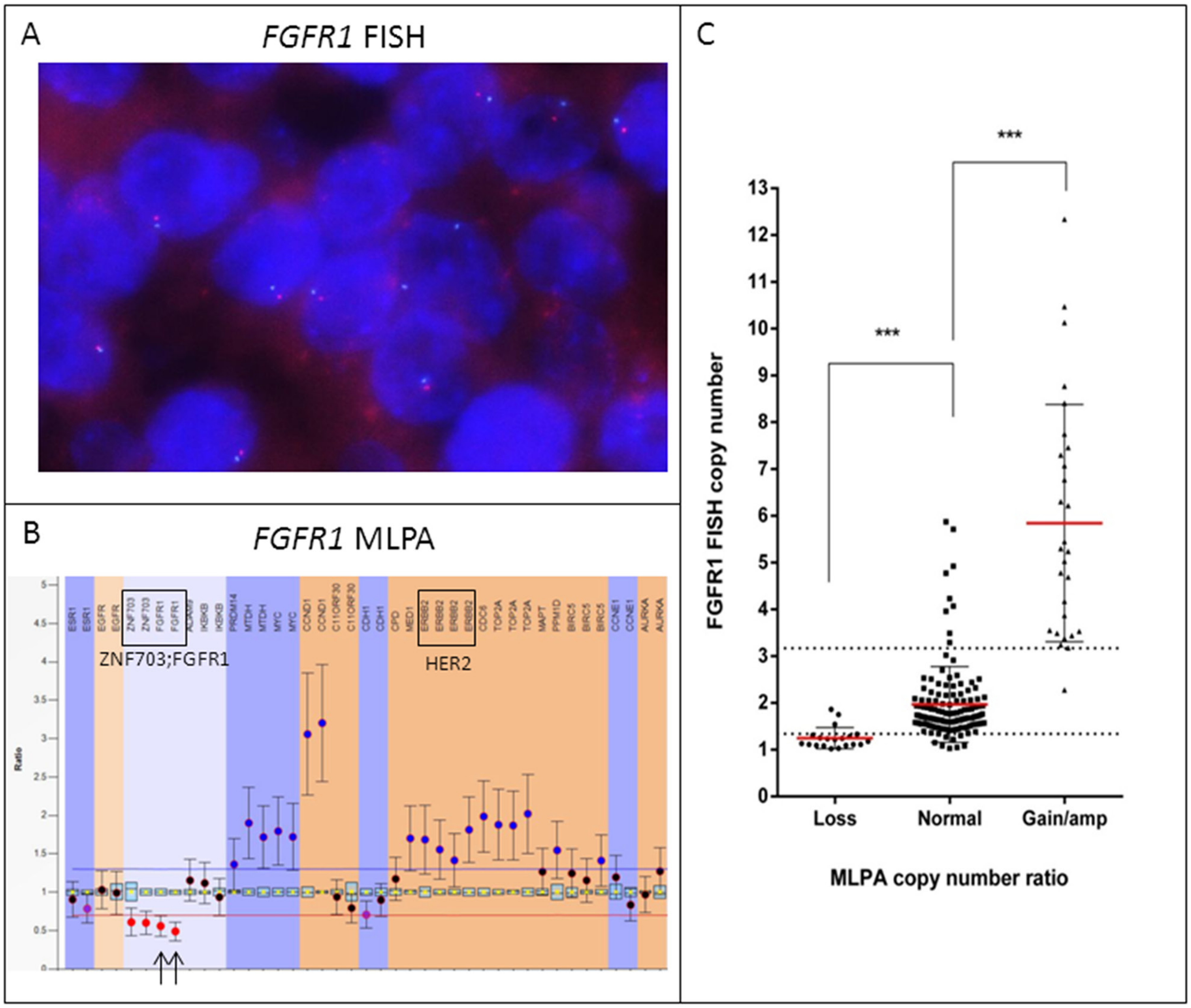

Supplementary Figure 3: *FGFR1* copy number correlation between MLPA and FISH. (A) *FGFR1* FISH loss. Red = *FGFR1*. Blue = CEP8 (green signal not shown). **(B)** MLPA showing *FGFR1* copy number loss (arrows) and *ZNF703* loss in association with *HER2* gain/amplification **(C)** Absolute *FGFR1* FISH copy numbers within each MLPA category (*** significant by ANOVA and Bonferroni posthoc analysis).

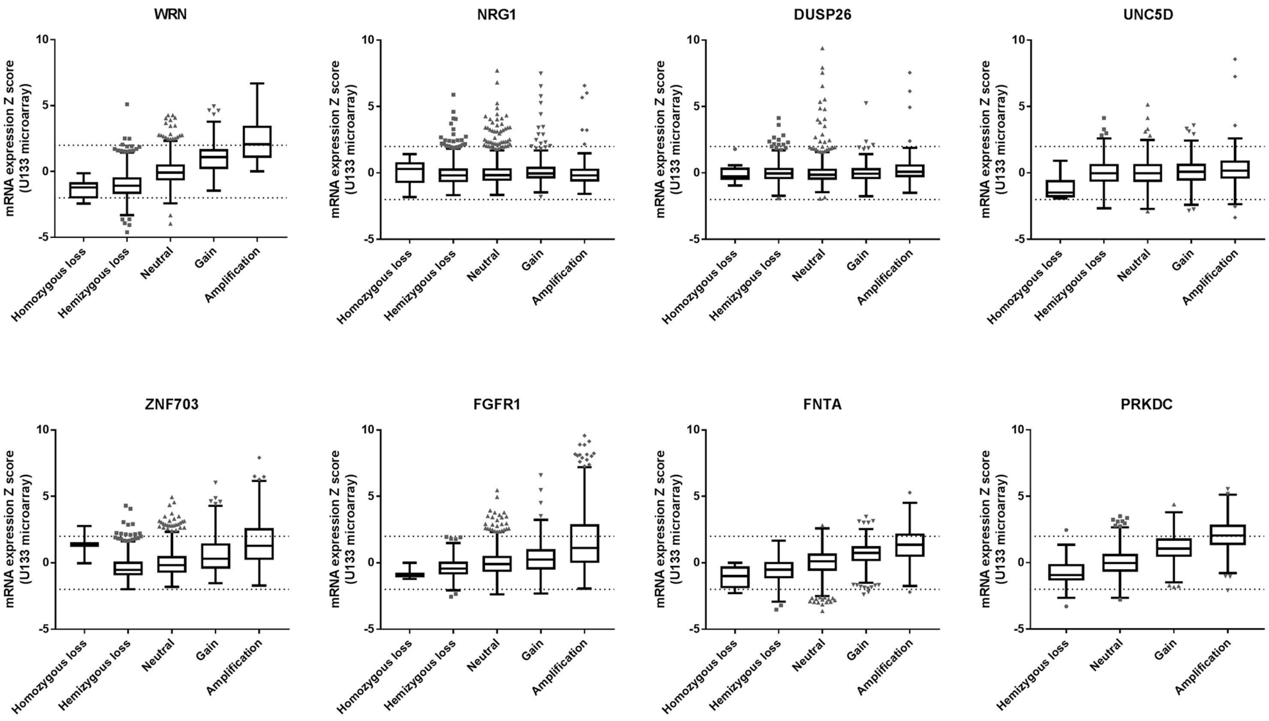

Supplementary Figure 4: Box and Whisker plots (Tukey method) illustrating correlation between METABRIC 8p11-12 copy number alterations and mRNA expression Z-scores. Horizontal lines indicate Z-scores of 2 and -2. Samples with Z-score >10 are not shown.

Supplementary Table 1: Sequences of the chromosome 8p11-12 MLPA probes and their locations

Gene	Genomic location	Ensembl cytogenetic band	Exon	Partial sequence (24 nt adjacent to ligation site)
**WRN**	31,033,262 bp from pter	8p12	9	ACTGGGGGAGTA-CAACAGAAACAA
			21	TTCGCCAAGTCA-TTCATTACGGTG
**NRG1**	31,639,304 bp from pter	8p12	2	GATTCAAGTGGT-TCAAGAATGGGA
			6	CTGGGACAAGCC-ATCTTGTAAAAT
**DUSP26**	33,591,330 bp from pter	8p12	2	CTGTTCGAACTC-GAGGGACCCTGG
			3	CCAGACGGCTGC-CGACTTCATCCA
**UNC5D**	35,235,457 bp from pter	8p12	5	GCTGACCATAAC-CTGATCATCAGG
			10	GTCTTTGGCCAT-TTAGGGGGGCGC
**ZNF703**	37,695,751 bp from pter	8p11.23	2	CACTTTGGGCCT-AAGCCGGTACCA
**FGFR1**	38,411,138 bp from pter	8p11.23	4	CAACCTCTAACT-GCAGAACTGGGA
			17	TGCATACACCGA-GACCTGGCAGCC
**FNTA**	43,034,194 bp from pter	8p11.21	6	GACCAACTTCTG-AAAGAGGATGTG
**PRKDC**	47,773,108 bp from pter	8q11.21	6	GGTGAAGTTCAT-CCTAGTGAGATG

Chromosomal region by ENSEMBL.

Supplementary Table 2: Clinical and pathological characteristics of the patients and primary tumors studied.

See Supplementary File 1

Supplementary Table 3: Basic clinical and pathological characteristics of the METABRIC dataset studied.

See Supplementary File 2

Supplementary Table 4: Raw chromosome 8p MLPA and corresponding clinical/pathological data.

See Supplementary File 3

Supplementary Table 5: Adjusted hazard ratios of 8p11-12 genes and (sub)regions significantly associated with event free survival in different treatment categories.

See Supplementary File 4

Supplementary Table 6: Comparison of MLPA and METABRIC copy number variation frequencies.

See Supplementary File 5

Supplementary Table 7: Associations between METABRIC chromosome 8 copy number variations and age, histological grade, tumor size, tumor stage, mutation load, ER/PR/HER2 status and 3-gene classifier-based intrinsic subtype.

See Supplementary File 6

Supplementary Table 8: Adjusted HR of 8p genes and (sub)regions significantly associated with disease free survival in different treatment categories of the METABRIC dataset.

See Supplementary File 7
